# Astragalin Inhibits the Proliferation and Migration of Human Colon Cancer HCT116 Cells by Regulating the NF-κB Signaling Pathway

**DOI:** 10.3389/fphar.2021.639256

**Published:** 2021-04-19

**Authors:** Min Yang, Wen-Yun Li, Jing Xie, Zi-Lin Wang, Yan-Long Wen, Cun-Chao Zhao, Liang Tao, Ling-Fei Li, Yang Tian, Jun Sheng

**Affiliations:** ^1^ College of Food Science and Technology, Yunnan Agricultural University, Kunming, China; ^2^ National Research and Development Professional Center for Moringa Processing Technology, Yunnan Agricultural University, Kunming, China; ^3^ Engineering Research Center of Development and Utilization of Food and Drug Homologous Resources, Ministry of Education, Yunnan Agricultural University, Kunming, China; ^4^ Key Laboratory of Pu-er Tea Science, Ministry of Education, Yunnan Agricultural University, Kunming, China; ^5^ Yunnan Province Engineering Research Center of Functional Food of Homologous of Drug and Food ,Yunnan Agricultural University, Kunming, China

**Keywords:** astragalin, colon cancer HCT116 cells, cell proliferation and migration, NF-κB signaling pathway, apoptosis and cell cycle arrest, inflammatory cytokines

## Abstract

Astragalin is a flavonoid found in a variety of natural plants. It has anti-inflammatory, anti-oxidant effects and has inhibited effects against several malignant tumor cell types. However, its effects on colon cancer and the molecular mechanisms have remained to be elucidated. In this study, we evaluated the inhibitory effect of astragalin on proliferation and migration of human colon cancer HCT116 cells *in vitro* and *in vivo*. Furthermore, we elucidated the mechanism of these effects. The results showed that astragalin significantly inhibited the proliferation and diffusion of HCT116 cells by induced apoptosis (by modulation of Bax, Bcl-2, P53, caspase-3, caspase 6, caspase 7, caspase 8, caspase 9 protein express) and cell cycle arrest (by modulation of Cyclin D1, Cyclin E, P21, P27, CDK2, CDK4 protein express). Moreover, astragalin suppressed HCT116 cell migration by inhibiting the expression of matrix metalloproteinases (MMP-2, MMP-9). In addition, astragalin significantly downregulated the expression of key proteins in the NF-κB signaling pathway and inhibited the transcriptional activity of NF-κB P65 stimulated with inflammatory cytokines TNF-α, thereby inhibiting the growth of colon cancer cells *in vitro*. Our further investigations unveiled astragalin gavage significantly reduced the proliferation of colon cancer xenograft in nude mice, *in vivo* experiments showed that tumor growth was related to decreased expression of apoptotic proteins in tumor tissues and decreased activity of the NF-κB signaling pathway. In summary, our results indicated that astragalin inhibits the proliferation and growth of colon cancer cells *in vivo* and *in vitro* via the NF-κB pathway. Therefore, astragalin maybe become a potential plant-derived antitumor drug for colon cancer.

## Introduction

Colon cancer is one of the most common malignant tumors and the second leading cause of cancer deaths (Park, et al., 2018). Colorectal cancer has a high morbidity rate (Sun and Zhu, 2018). According to the World Health Organization, there were 18.1 million new colon cancer cases worldwide in 2018; by 2030, the worldwide burden is expected to increase by 60% colorectal cancer total number (Zhang Z. H. et al., 2020). Colon cancer seriously affects patient quality of life. At present, in addition to surgery, chemotherapy and radiotherapy are the two main strategies for the treatment of colorectal cancer. However, these treatments have certain side effects, including nausea, diarrhea, and low quality of life (Li K. et al., 2017). In recent decades, significant progress has been made in identifying therapeutic targets and exploring potential therapeutic compounds, there are about 61% of anticancer compounds, and 49% of anti-infection compounds are from natural products (Luo et al., 2014). Many research efforts have focused on the development of natural anticancer drugs. In particular, active compounds in natural functional foods are of interest as potential anticancer drugs.

The nuclear factor-κB (NF-κB) signaling pathway as a key player was involved inflammation-induced tumor metastasis (Zhang L. et al., 2020). The inflammatory response regulated by pro-inflammatory NF-κB transcription factors plays a crucial part in the development of colorectal cancer. NFκB consists of P65 and P50 in normal cells, with an inhibitory IκBα subunit active (Karin, 2006). It remains in an inactive state in most cancers, activation of IκB kinase (IKK) results in IκBα phosphorylation and targeting of ubiquitin, the P65 and P50 subunits are released and migrate to the nucleus, where they combine with specific DNA sequences to activate NFκB pathways (Gupta et al., 2010). Besides, many inflammatory mediators in tumor microenvironment to tumor progression (Jing and Lee, 2014). Tumor inflammatory cytokines cells could produce tumor necrosis factor-α (TNF-α) (Li Y. et al., 2019), interleukin-6 (IL-6) (Wouters et al., 2014), and other inflammatory factors, which can active several signal pathways, such as NF-κB pathway (Gray et al., 2014; Garbers et al., 2015). These Inflammatory factors modulate tissue remodeling and promote tumor cell migration through autocrine and paracrine (Liu et al., 2018). Activated NF-κB exists in various colorectal cancer cell lines, xenograft animal models, and human colorectal cancer tissues (Voboril and Weberova-Voborilova, 2006; Kim S. et al., 2008). NF-κB pathway activation mediates cell proliferation (Thoms et al., 2007), cell death, cell invasion, metastasis (Ban et al., 2007) and angiogenesis via protein expression to support the growth of colorectal cancer (Yu et al., 2004; Sutnar et al., 2007). Thus, drugs that block activation of the NFκB pathway have potential uses in treating colorectal cancer.

**FIGURE 1:**
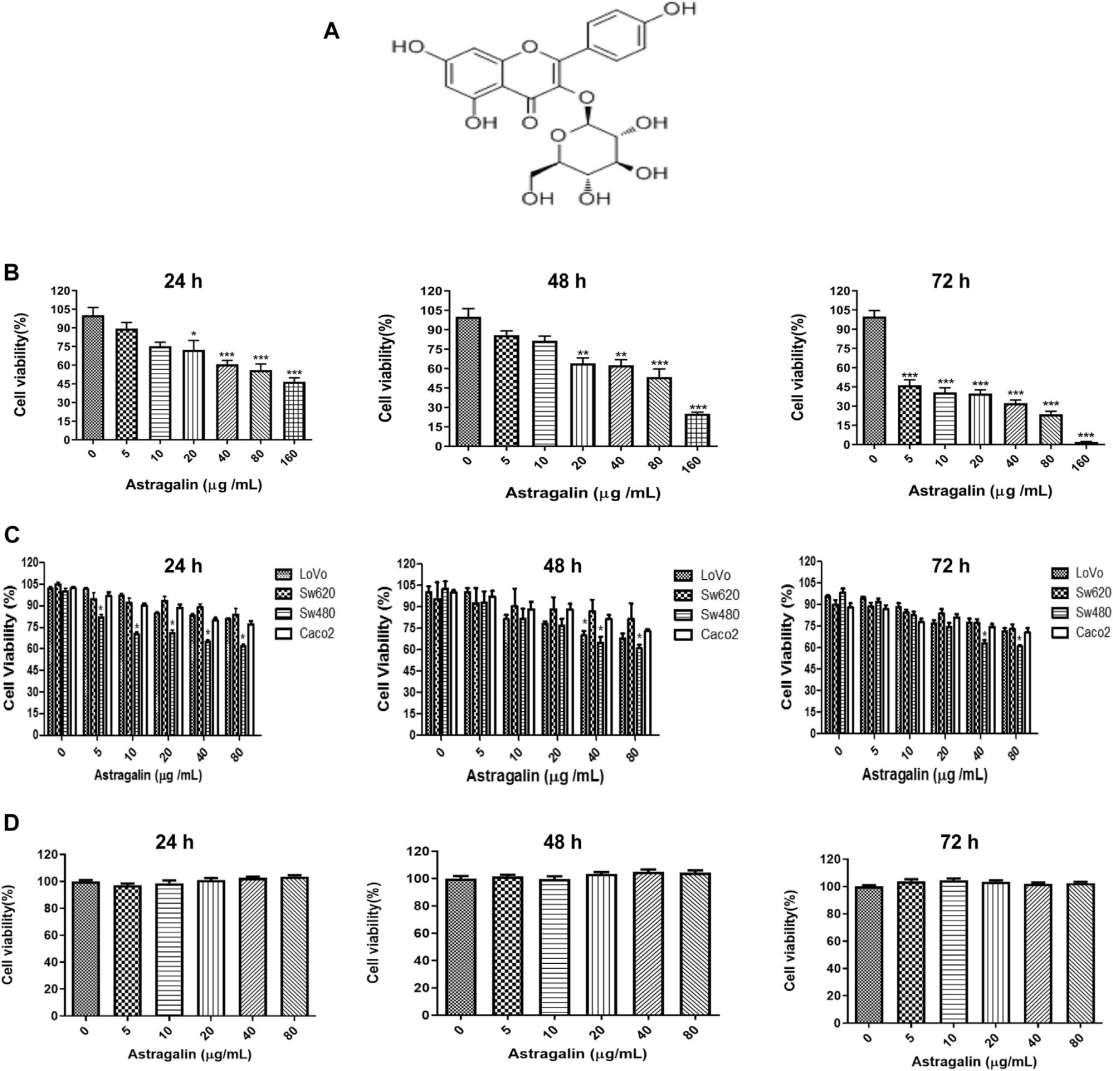
Astragalin inhibited the proliferation of human colon cancer cells. **(A)** Chemical structure of astragalin. **(B)** Inhibition of the proliferation of HCT116 colon cancer cells by different concentrations of astragalin. The IC_50_ values of astragalin in HCT116 cells. **(C)** Inhibition of the proliferation of LoVo, SW620, SW480, Caco2 cells by treatment with astragalin. **(D)** Effects of astragalin on cell viability of NCM460 cells. Data are expressed as mean ± SEM (*n* = 3); “*” indicates the difference between one group and the control group, respectively. *p < 0.05, **p < 0.01, ***p < 0.001 vs. 0.0 μg/ml.

Astragalin (ASG) (Figure 1A), also known as kaempferol-3-O-glycosidase, is a natural flavonoid found in various herbs and medicinal plants (Liu et al., 2019), including horseradish tree leaf, lotus leaf, and Chinese rose (Li et al., 2018; Oldoni et al., 2019; Li et al., 2020) as well as in some fruits and vegetables such as persimmon (Harikrishnan et al., 2020). Astragalin is used in various pharmacological applications owing to its anti-inflammatory, antioxidant, anticancer, bacteriostatic properties, its ability to provide nerve and heart protection, and resistance against and osteoporosis (Rey et al., 2019; Harikrishnan et al., 2020). For instance, astragalin can alleviate acute lung injury caused by lipopolysaccharide-induced inflammation and airway thickening in mice (Soromou et al., 2012; Kim et al., 2017), and it could also delay aging induced by superoxide dismutase and catalase D-galactose in mice (Li C. et al., 2019). Moreover, current cancer researches indicate that flavonoids could be used alone or in combination with other drugs to control the growth of various types of tumor cells. Astragalin can lower the expression of hexokinase, thereby inhibiting the growth of liver cancer (Li W. et al., 2017), inhibiting the proliferation of lung cancer and melanoma cells (Chen et al., 2017; You et al., 2017). However, the effects of astragalin on human colon cancer HCT116 cells and the molecular mechanisms through which astragalin exerts its impacts have remained to be fully elucidated. In this study, we examined the anticancer effects of astragalin on inhibiting the proliferation and migration of HCT116 cells, explored their potential molecular mechanism *in vitro* and *in vivo*.

## Materials and Methods

### Materials

Astragalin (purity ≥99%) was purchased from Dericke Biotechnology Co., Ltd. (Chengdu, Sichuan, China). MTT ((3-4,5-dimethylthiazole-z-yl)-3,5-diphenyltetr-azoliumbromide), trypsin, penicillin-streptomycin, DMSO, and PBS were purchased from Solaibao Technology Co., Ltd (Beijing, China) and stored at −20°C. TNF-α was purchased from Sigma (Louis, MO, United States). Fetal bovine serum was obtained from Gibco (Gaithersburg, United States). The annexin-V/PI apoptosis kit and BCA protein quantitative kit were acquired from Biyuntian Biotechnology Co., Ltd (Shanghai, China). Cell transwell plates were bought from Corning costar Inc. (NY, United States). Antibodies against Bax, Bcl-2, NF-κB, and p-NFκB were acquired from Cell Signaling Technology (Beverly, United States). The primary antibodies against caspase 3, cleaved caspase-3, caspase 6, caspase 7, caspase 8, caspase 9, CDK4, CDK2, Cyclin D1, Cyclin E, P53, P21, P27, NFκB P65, P-NFκB P65 were acquired from Proteintech Inc. (Rosemont, United States). Nunc™ Lab-Tek™ II Chamber Slide™ were obtained from Thermo Fisher Scientific Inc. (NY, United States). Four-week-old male BALB/c nude mice were bought from Kevins Animal Laboratory Co., Ltd. (Changzhou, China) and maintained in a specific-pathogen-free (SPF) environment with adequate water and food.

### Cell Lines and Culture

Human colon cancer cell lines (HCT116, LoVo, SW620, SW480, Caco2) and human colon epithelial cell lines NCM460 were obtained from the Kunming Cell Bank of the Typical Culture Preservation Committee of the Chinese Academy of Sciences. HCT 116, SW620, SW480, and LoVo cells were cultured in DMEM/F12 medium. Caco2 cells were cultured in DMEM/RPMI 1640 medium. NCM 460 cells were cultured in DMEM High medium (HyClone, CA, United States). All cell types were cultured in a humidified atmosphere with 5% CO_2_ at 37°C. All media were supplemented with 10% FBS and a 1% penicillin-streptomycin mixture.

### MTT Cell Viability Assay

Five human colon cancer cell lines (HCT116, LoVo, SW620, SW480, Caco2) in the logarithmic growth phase were seeded in 96-well plates, adjusted to a concentration of 2 × 10^4^ cells per well. After culturing for 24 h, cells were treated with different concentrations of astragalin (5, 10, 20, 40, and 80 μg/ml). Next, the cells were incubated for 24, 48, 72 h, then 5 μg/ml MTT was added to each well. After incubation for a further 4 h, the medium was replaced with DMSO, and OD values were measured at wavelength 490 nm by a microplate reader.

The HCT116 cells and human colon epithelial cell lines NCM460 (2 × 10^4^) were seeded in 96-well plates for 24 h and treated with different concentrations of astragalin (5, 10, 20, 40, 80, 160 μg/ml) for 24, 48, 72 h. The cell viability was evaluated by MTT assay. The half of maximal inhibitory concentration (IC_50_) values of HCT116 cells were calculated as the drug concentrations necessary to inhibit 50% proliferation of cells.

The cell inhibition rate was calculated by the following equation:

$$\begin{array}{l} \text{cell}\;\text{inhibition}\;\text{rate}\;\text{of}\;\text{colon}\;\text{cancer}\;\text{proliferation }=\;1-(\text{OD}\;\text{value}\;\text{of}\;\text{the}\;\text{drug}\;\text{treatment}\\ \text{group}/\text{OD}\;\text{value}\;\text{of}\;\text{the}\;\text{cell}\;\text{control}\;\text{group})\text{× }100\text{\%}\text{.} \end{array}$$

### Cell Colony Assay

HCT116 cells were incubated in six-well plates then treated with astragalin (20, 40, or 80 μg/ml) for different times (24, 48, or 72 h). After culturing for 14 days, the cells were washed three times with PBS, then fixed with 4% paraformaldehyde solution for 10 min before being stained with 0.1% crystal violet for 30 min. After the staining solution was removed, cells were dried, photographed, and counted, and the cell colony formation rate was calculated as follows:

cell colony formation rate=cell colony number/number of cultured cells×100%

### Cell Migration Analysis

HCT116 cells were cultured in six-well plates at 3,000 cells per well. Two hundred microlitres pipette perpendicular to the orifice plate from top to bottom was used to make scratches. The medium was discarded, and astragalin was added at different concentrations. After 48 h, cells were washed twice with PBS. Finally, the numbers of migrating cells in each group were calculated using the *ImageJ Pro* software.

### Cell Transwell Migration Analysis

HCT116 cells were cultured in 100 µL of serum-free medium with astragalin in the top chambers of a 24-well transwell plate, at a concentration of 1 × 10^4^ cells per well, while 600 µl medium with 20% FBS was added to the lower chambers. The plate was incubated in 5% CO_2_ for 24 h at 37°C. Penetration of cells through the porous membrane was detected by crystal violet staining and observed with a light microscope (Olympus). Numbers of stained cells were counted in six random fields of each plate chamber.

### Cell Apoptosis and Cell Cycle Analysis

HCT116 cells (9 × 10^3^ cells per well) were inoculated in a six-well plate for 24 h. After treatment with four different concentrations of astragalin (0, 20, 40, and 80 μg/ml) for 48 h, cells were washed three times with cold PBS. For the cell apoptosis assay, the incubated cells were collected and stained with an annexin-V/propidium iodide (PI) apoptosis kit. Cells were resuspended in 100 µl 1X binding buffer containing 5 µl FITC annexin V and 5 µl PI and incubated for 30 min in the dark. Apoptosis of HCT116 cells was detected and analyzed by flow cytometry. For the cell cycle analysis, cells were harvested and fixed in 70% ethanol and stored at −20°C overnight. The cells were then washed twice with PBS to remove ethanol residue and stained with 0.5 ml PI/RNase staining buffer in the dark at room temperature for 30 min. The cell cycle was analyzed by flow cytometry and *FlowJo 9.0*.

### NF-κB P65 Nuclear Translocation Analysis

HCT116 cells were inoculated in a six-well plate for 24 h. Then cells were treated with astragalin (80 μg/ml) for 4 h or 8 h. Before the treatment time, cells were indicated with 20 ng/ml TNF-α for 1 h. Treated cells were washed with cold PBS three times, then were fixed in 4% paraformaldehyde for 30 min at room temperature, washed three times with PBS, and permeabilized with 0.2% Triton X-100 (Sigma, Louis, MO, United States) for 10 min at room temperature, washed with PBS and blocked with 5% PBS for 1 h. Antibody P65 was added in 1% BSA (1:200) and incubated cells overnight at 4°C. After washing with PBS three times, then incubated with a secondary antibody goat - anti-rabbit IgG (1:200, Cell Signaling Technology, Beverly, United States) for 30 min at room temperature. Then DAPI was added to stain the cell nucleus. Fluorescence cells were observed and photographed under a confocal fluorescence microscope (Olympus, FV 1000).

### Enzyme Linked Immunosorbent Assay (ELISA)

HCT116 cells were inoculated in a six-well plate for 24 h and treated with astragalin (80 μg/ml) for 48 h. MMP-2, MMP-9, TNF-α, and IL-6 released from cell culture supernatants were investigated by an ELISA kit (Mei Mian Biotechnology Co., Ltd., Jiangsu China) according to the kit instructions. The color reaction was developed by chromogenic agent and the optical density was measured at *λ* = 495 nm by micro plate reader.

### *In Vivo* Animal Tumor Model

In order to determine the effects of astragalin on colon cancer *in vivo*, 4-week-old male BALB/c nude mice were fed in an SPF environment. HCT116 cells (5 × 10^6^/ml) in 200 µl PBS were injected subcutaneously into the flank of each mouse. By 12 days, the tumor volumes had grown to 100–120 mm^3^. All nude mice were then randomly divided into four groups: control group (*n* = 5, 0 mg/kg astragalin); low-dose group (*n* = 5, 25 mg/kg astragalin); medium-dose group (*n* = 5, 50 mg/kg astragalin); high-dose group (*n* = 5, 75 mg/kg astragalin). The drug was administered by gavage every 2 days. The formula v = 1/2 ab^2^ (a as length, b as width) was used to calculate tumor volumes. Body weights and total food intake of mice were measured every 4 days. The formula Food or Water intake = food add over the first day − food left over the fourth day (then add the food to the average as the first given). After 25 days of treatment, the mice were sacrificed and dissected, and tumor tissues were weighed and fixed in 4% formalin for 24 h. The animal experiment was reviewed and approved by the Yunnan Agricultural University Animal Ethics Committee (YNAU-2020-011).

### Tumor Immunohistochemistry

Samples of tumor tissue were removed from formalin and embedded in paraffin. Expression levels of NF-κB in tissue were determined by immunohistochemical analysis. Slides were treated with 3% hydrogen peroxide and incubated with NF-κB (1:200) antibodies, then incubated with anti-goat IgG (1:500) secondary antibody (Jackson ImmunoResearch, United States) for 30 min at room temperature. Slides were washed, stained with DAB, counterstained with hematoxylin, and sealed after dehydration. All images were captured using an inverted fluorescence microscope (Nikon, Japan) and analyzed by *Image J*.

### Western Blotting

Cells after treatment with astragalin (80 μg/ml) for 48 h were detected the cell cycle and apoptosis. HCT116 cells were stimulated with astragalin (80 μg/ml) for variable durations (between 0.5 and 8 h) to observed the phosphorylation of the NF-κB pathway at a short time incubated. Cells were treated with 20 ng/ml TNF-α for 2, 4, and 8 h to investigated the effects of TNF-α induced NF-κB phosphorylation. The TNF-α was used as a pretreatment for 1 h which was followed by 4 h or 8 h of astragalin (80 μg/ml), then used nuclear and cytoplasmic protein extraction kit (Beyotime, Shanghai, China) to the separation of cell nuclear and cytoplasmic for the protein level of NF-κB p65 in nuclear and cytoplasmic.

Cell total protein was extracted by RIPA buffer (Beyotime, Shanghai, China), protein concentrations were assessed by the BCA assay kit. Target protein samples were separated by 10% SDS-PAGE and transferred by electroblotting to polyvinylidene difluoride membranes (Millipore, MA, United States). After blocking with 5% skim milk for 2 h, proteins were incubated with primary antibodies against Bax, Bcl-2, P53, caspase 3, cleaved caspase 3, caspase 6, caspase 7, caspase 8, caspase 9, CDK4, CDK2, Cyclin D1, Cyclin E, NF-κB, IKKα/β and β-actin overnight at 4°C. The PVDF membranes were then washed three times with TBST (Tris Hcl Tween) for nearly 8 min each time, then incubated for 1 h with goat -anti -rabbit -HRP or goat -anti -mouse -HRP (1:10000, Abcam, MA, United States). The signal was detected using ECL western blotting substrate and analyzed by *Image J*.

Tumor tissues were collected, and the protein of the NF-κB signaling pathway and β-actin were determined by western blotting.

### Statistical Analysis

The experimental data were analyzed using GraphPad Prism 7.0. Values are expressed as mean ± standard error of the mean (SEM). Results comparisons were used one-way analysis of variance (ANOVA). *p < 0.05, **p < 0.01, and ***p < 0.001; ^**#**^ *p* < 0.05, ^**##**^ *p* < 0.01, and ^**###**^ *p* < 0.001 were considered to indicate statistically significant differences.

## Results

### Effects of Astragalin on the Growth of Colon Cancer Cells *in Vitro*

To determine the anti-cancer effect of astragalin, we used four different types of human colon cancer cell lines (HCT116, LoVo, SW620, SW480, Caco2) were treated with astragalin (0, 5, 10, 20, 40, and 80 μg/ml). The MTT assay showed that after treatment for 24, 48, or 72 h, astragalin had a significant inhibitory effect on human colon cells, especially inhibited on HCT116 cells and gradually increased with increasing drug concentrations (Figures 1B,C). IC_50_ values of the HCT 116 were 121.845, 87.908, and 18.883 μg/ml at 24, 48, and 72 h that treated with astragalin (0, 5, 10, 20, 40, 80, and 160 μg/ml). To further investigate the effect of astragalin on the nonmalignant human colon epithelial cells, NCM460 cells were treated with astragalin (0, 5, 10, 20, 40 and 80 μg/ml) for 24, 48, and 72 h. The results showed that astragalin has a strong inhibitory effect on HCT116 human colon cancer cells but no toxicity or effects on proliferation in normal colon cells (NCM460) (Figures 1B,D). Respectively, based on these results, we selected astragalin concentrations of 20, 40, and 80 μg/ml for the subsequent experiments.

### Astragalin Inhibited the Migration of HCT116 Cells

**FIGURE 2:**
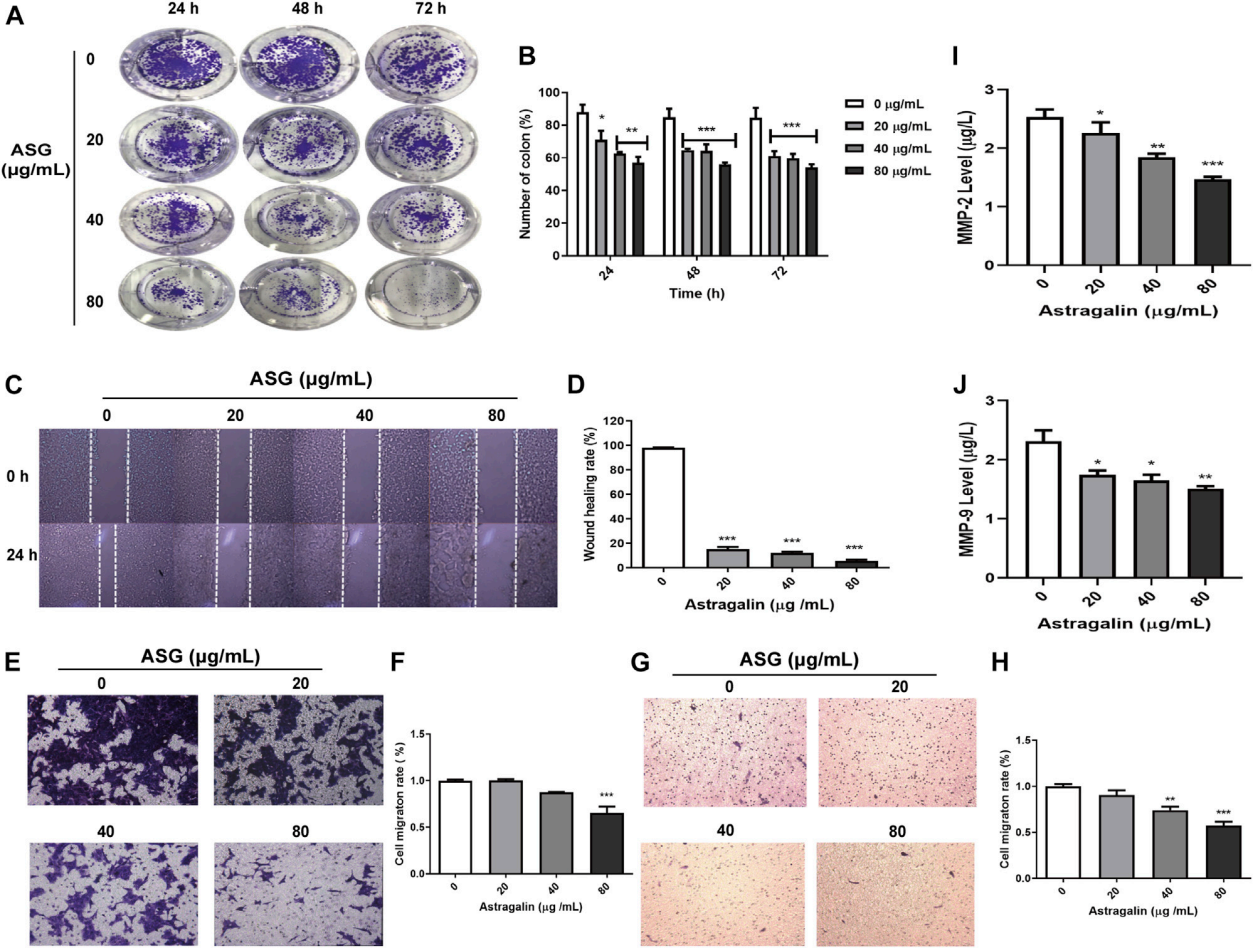
Astragalin inhibited the migration of HCT116 cells with effects on migration and invasion. **(A,B)** Colony experiment showing effects of astragalin on HCT116 cells. **(C,D)** Wound healing assay in HCT116 cells showing inhibition by astragalin (scratch analysis). **(E,F)** Transwell invasion assay showing that astragalin inhibited migration of HCT116 cells. **(G,H)** Astragalin inhibited migration of HCT116 cells by transwell migrate assay. **(I,J)** expression levels of MMP-9 and MMP-2 as determined by Elisa. Data are expressed as mean ± SEM from three independent experiments (*n* = 3). *p < 0.05, **p < 0.01, ***p < 0.001 vs. 0.0 μg/ml.

To explore the mechanism underlying the inhibited effects of astragalin on HCT116 cells, we used HCT116 cells to conduct cloning experiments, cell scratch assays, and cell migration assays. HCT116 cells were inoculated with astragalin in six-well plates until the cell colonies were visible and could be counted. The colony formation rate of the drug group was significantly different from that of the control group (*p* ≤ 0.001). Moreover, with increasing time and drug concentration, the number of cell colonies decreased significantly, with a cloning inhibition rate of about 50.2% at 80 μg/ml (Figures 2A,B). In order to observe the migration ability of cells, we used cell scratch experiments to measure the migration distance of cells. To avoid cells of the control group becoming connected in clusters we selected 24 h as the experiment duration. The results showed that astragalin inhibited the movement of HCT116 cells in a dose-dependent manner, and the scratching width of the cells in the treatment group was significantly greater than that of the control group after 24 h. Compared with the control group, HCT116 cells treated with 20, 40, and 80 μg/ml concentrations of astragalin showed mobilities of 14.38, 13.44, and 5.29%, which represented a significant effect (*p* < 0.001) (Figures 2C,D). Transwell assay showed that the number of membrane penetrations of HCT116 cells decreased significantly after treatment (Figures 2E–H). ELISA was used to detect the release of MMP-2 and MMP-9 enzymes to the HCT 116 cells culture medium, and showed that the release of MMP-2 and MMP-9 enzymes was significantly reduced after treatment with astragalin **(** Figures 2I,J **)**. Thus, we conjectured that the inhibition of invasion and migration of colon cancer cells by astragalin might be related to the downregulation of matrix metalloproteinases (MMPs).

### Astragalin Promotes Apoptosis of HCT 116 Cells

**FIGURE 3:**
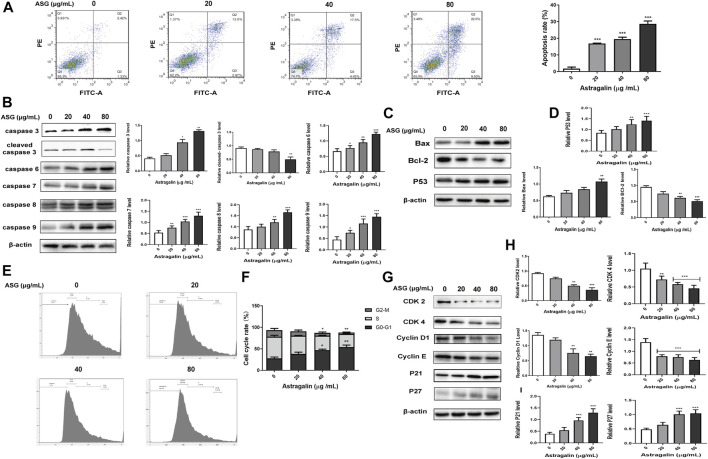
Astragalin promoted apoptosis and cell cycle arrest in colon cancer HCT116 cells. **(A)** The apoptosis test group of HCT116 cells treated with astragalin. **(B–D)** Expression of apoptotic proteins (caspase 3, cleaved caspase 3, caspase 6, caspase 7, caspase 8, caspase 9, Bax, Bcl2, and P53) in HCT116 cells. **(E,F)** Cells were exposed to different concentrations of astragalin, and the cell cycle was analyzed by flow cytometry. **(G–I)** Protein expression levels of CDK2, CDK4, Cyclin D1, Cyclin E, P21, and P27 determined by western blotting. Data are expressed as mean ± SEM from three independent experiments (*n* = 3). *p < 0.05, **p < 0.01, ***p < 0.001 vs. 0.0 μg/ml.

To determine whether the inhibitory effect of astragalin on colon cancer cells was related to the regulation of cell apoptosis, annexin V-FITC/PI staining was used to detected cell apoptosis by flow cytometry. After treatment of cells with astragalin for 48 h, the apoptotic cell population was markedly increased compared with that of the control group, the proportions of early and late apoptotic cells in the 0, 20, 40, and 80 μg/ml concentration groups were 8.37%, 16.85% (*p* < 0.001), 19.5% (*p* < 0.001) and 28.6% (*p* < 0.001) (Figure 3A), respectively. We also evaluated the expression levels of apoptotic protein in HCT116 cells by western blotting. It showed that astragalin increased the expression of pro-apoptotic proteins (caspase 3, caspase 6, caspase 7, caspase 8, caspase 9, P53, and Bax) and decreased the expression of anti-apoptotic proteins (cleaved caspase-3 and Bcl-2) in a dose-dependent manner **(** Figures 3B–D).

### Astragalin Induced G0/G1 Cell Cycle Arrest of HCT116 Cells

Next, the cell cycle distribution of HCT116 cells was examined after treatment with different concentrations of astragalin for 48 h. Compared with the control group, astragalin treatment increased the proportion of G0/G1-phase cells. The proportions of cells in G0/G1 phase in the 0, 20, 40, and 80 μg/ml concentration groups were 27.56, 38.24, 47.04% (*p* < 0.001), and 53.86% (*p* < 0.001), respectively, those of cells in S phase were 50.46, 42.16, 33.36% (*p* < 0.05), and 30.56% (*p* < 0.001) (Figures 3E,F), respectively, the accumulation of S-phase cells was also significantly reduced by increasing concentrations of astragalin. These results indicate that astragalin blocked the growth of HCT116 cells, in particular G0/G1-phase growth. To further investigate the potential anticancer mechanism of astragalin in HCT116 cells, we used western blotting to detect the expression of essential proteins that regulate the progression of the cell cycle. Furthermore, the results showed astragalin significantly decreased the expression levels of CDK2, CDK4, Cyclin D1, Cyclin E and increased the expression of P21 and P27. both of protein which regulates G0/G1-phase cells (Figures 3G–I). These results suggested that astragalin inhibited cell proliferation by inducing cell cycle arrest in HCT116 cells.

### Astragalin Inhibited HCT116 Cell Proliferation via the NF-κB Pathway *in Vitro*

**FIGURE 4:**
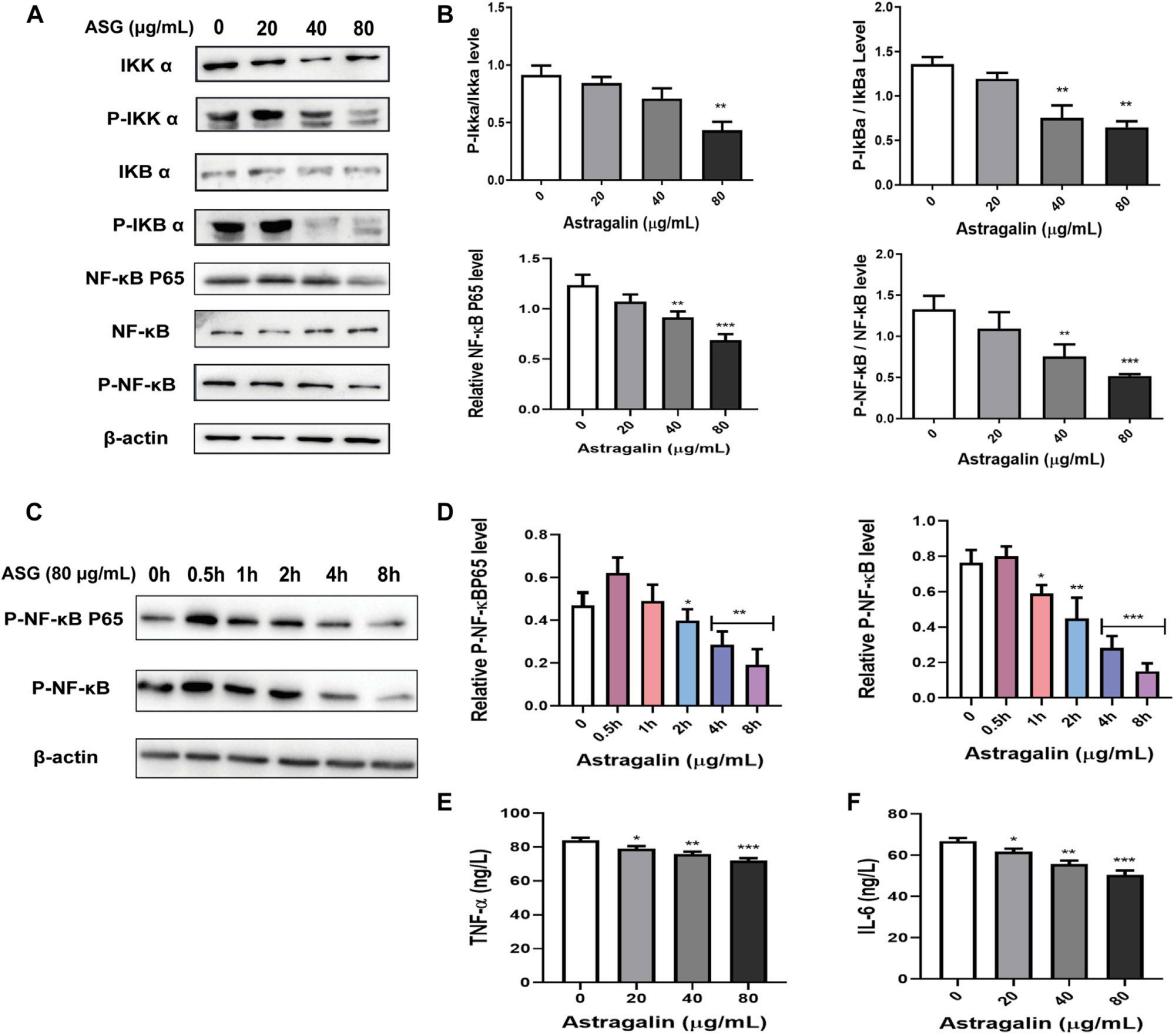
Astragalin inhibited NF-κB signaling pathways. **(A,B)** Protein expression of Iκκα, p-Iκκα, IκBα, p-IκBα, NF-κB, p-NF-κB, NF-κB P65 determined by western blot after cells were exposed to astragalin for 48 h. **(C,D)** Protein expression of p-NF-κB and P-NF-κB P65 determined by western blot after cells were exposed to astragalin for a short time treatment. **(E,F)** expression levels of TNF-α and IL-6 as determined by Elisa. Data are expressed as mean ± SEM from three independent experiments (*n = 3*). *p < 0.05, **p < 0.01, ***p < 0.001 vs. 0.0 μg/ml.

To further clarify the mechanisms underlying astragalin medicated inhibition of growth and migration of HCT116 cells, the expression phosphorylated and unphosphorylated key proteins levels of NF-κB signaling pathway were assessed using western bolting. The results showed that treatment with astragalin decreased the expression of p-NF-κB and NF-κB P65 in a dose-dependent manner, suggesting that the effects of astragalin on HCT116 cells may be related to the NF-κB signaling pathway (Figures 4A,B). Then we investigated whether the inhibition of NF-κB activation by astragalin was related to the inhibition of IκBα phosphorylation, it showed the expression of *p*-IκBα was also inhibited. As the Iκκ protein is necessary for IκBα phosphorylation, astragalin can inhibit IκBα phosphorylation (Figures 4A,B). Furthermore, to evaluate the fast process of phosphorylation on protein, we observed the phosphorylation of NF-κB pathway protein at a different time point. We used astragalin (80 μg/ml) incubating the HCT116 cells for a few hours (0.5, 1, 2, 4 and 8 h). It could find significant attenuation of p-NF-κB, and p- NF-κB P65 after 4 h of astragalin treatment in a time-dependent manner (Figures 4C,D). Then we used an ELISA kit to determine the levels of inflammatory cytokines (TNF-α, IL-6) in the supernatant medium of astragalin-treated in HCT116 cells. The results showed that astragalin at 20, 40, and 80 μg/ml significantly reduced the level of TNF-α and IL-6 in cell supernatant (Figures 4E,F). These data suggested that astragalin could inhibit the secretion of inflammatory cytokines from human colon cancer cell HCT116.

### Astragalin Interrupts the NF-κB Signaling Pathway and Inhibits NFκB P65 Transcriptional Activity in HCT116 Cells Stimulated by TNF-α

**FIGURE 5:**
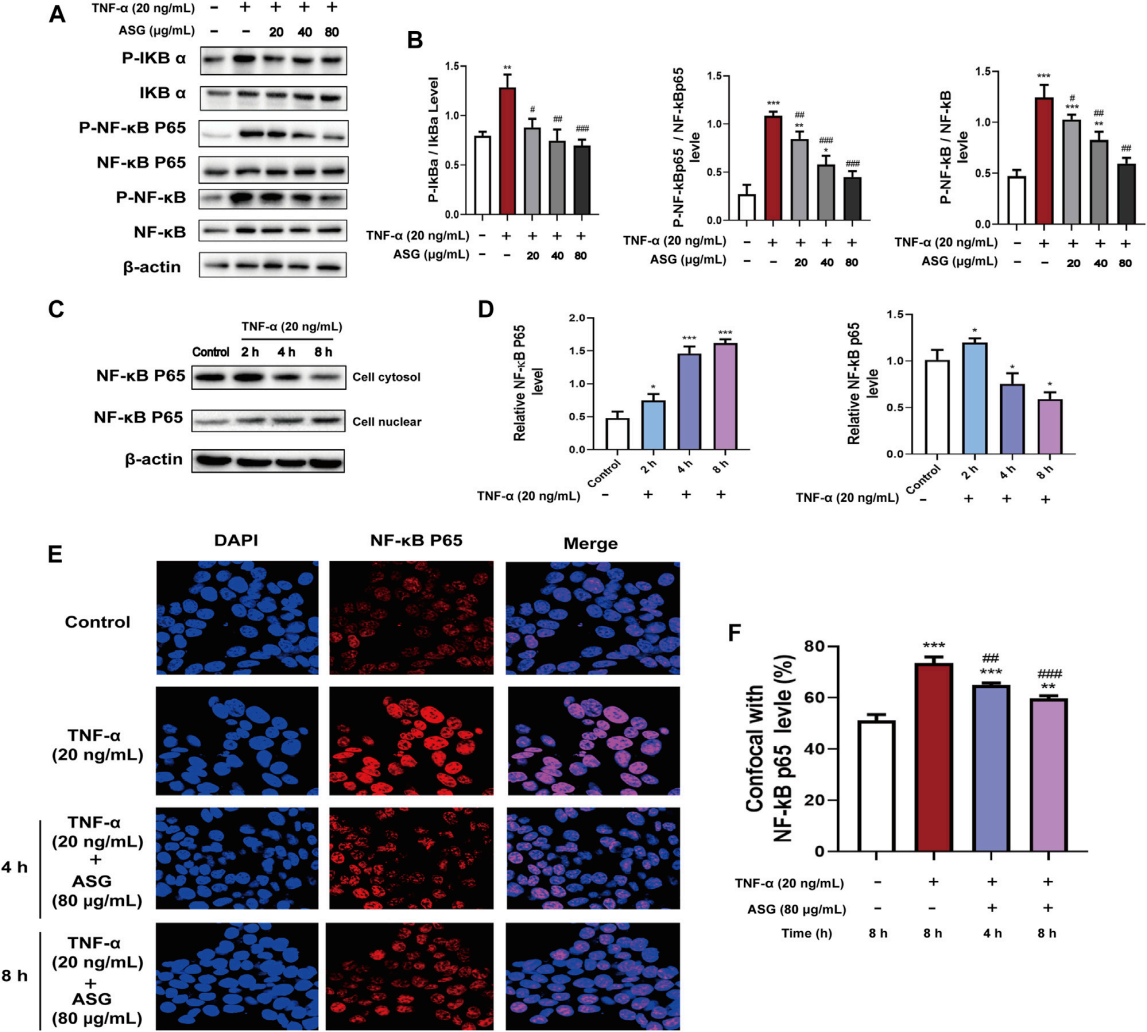
Astragalin inhibited NF-κB signaling pathways in HCT116 cells stimulated with TNF-α. **(A,B)** Protein expression of IκBα, p-IκBα, NF-κB, p-NF-κB, NF-κB P65, P-NF-κB P65 determined by western blot after cells were exposed to astragalin and TNF-α. **(C,D)** Protein expression of NF-κB P65 in the cytosol and nuclear determined by western blot after cells were stimulated with TNF-α. **(E,F)** Immunofluorescence confocal staining for determining NF-κB P65 nuclear translocation. Data are expressed as mean ± SEM from three independent experiments (*n = 3*). *p < 0.05, **p < 0.01, ***p < 0.001. vs. Control, ^#^ *p* < 0.05, ^##^ *p* < 0.01, ^###^ *p* < 0.001 vs. TNF-α group.

To further determine whether astragalin affected NF-κB Signaling Pathway that involved in cytokines inflammation-induced. The result showed that after TNF-α (20 ng/ml) treatment, the NF-κB Signaling Pathway was obviously activated, and the key protein levels of NF-κB signaling pathway like P-NFκB, P- IκBα, P-NFκB p65 were upregulated (Figures 5A,B). However, after treatment with astragalin (80 μg/ml) inhibited the expression levels of P-NFκB, P-IκBα, P-NFκB p65, indicating that the TNF-α induced NF-κB Signaling Pathway was blocked by astragalin (Figures 5A,B). Furthermore, to discuessed the effect of inflammatory cytokines TNF-α on the NF-κB Pathway and the nuclear translocation is also required for NF-κB signal transduction. Thus, we detected the cytosolic and nuclear levels of the NFκB p65 at different times. HCT116 cells were treated with TNF-α (20 ng/ml) for 2, 4, and 8 h. When following the time course of TNF-α induced NF-κB activation, the elevation of the NF-κB p65 in the cytosol of HCT116 cells after 2 h. The translocation of NF-κB p65 to the nucleus occurred after 4 h (Figures 5C,D). It indicated that the enhanced NF-κB p65 translocation into the nucleus was related to TNF-α. Besides, the nuclear translocation of the NF-κB p65 protein was also demonstrated by immunofluorescent staining. HCT116 cells were treated with TNF-α (20 ng/ml) for 1 h, followed for 4 and 8 h of astragalin (80 μg/ml) stimulation. Confocal fluorescence microscopy showed that the expression of NF-κB p65 in the nuclear of astragalin-treated HCT116 cells was lower than that in TNF-α-treated cells (Figures 5E,F). Therefore, it revealed that the enhanced NF-κB p65 translocation into the nucleus was abolished by astragalin in a time-dependent manner. These results collectively indicated that astragalin inhibited the transcriptional activity induced by TNF-α and suppressed NF-κB signaling pathway.

### Astragalin Inhibited Tumor Growth in Xenograft Nude Mouse Model *in Vivo*

**FIGURE 6:**
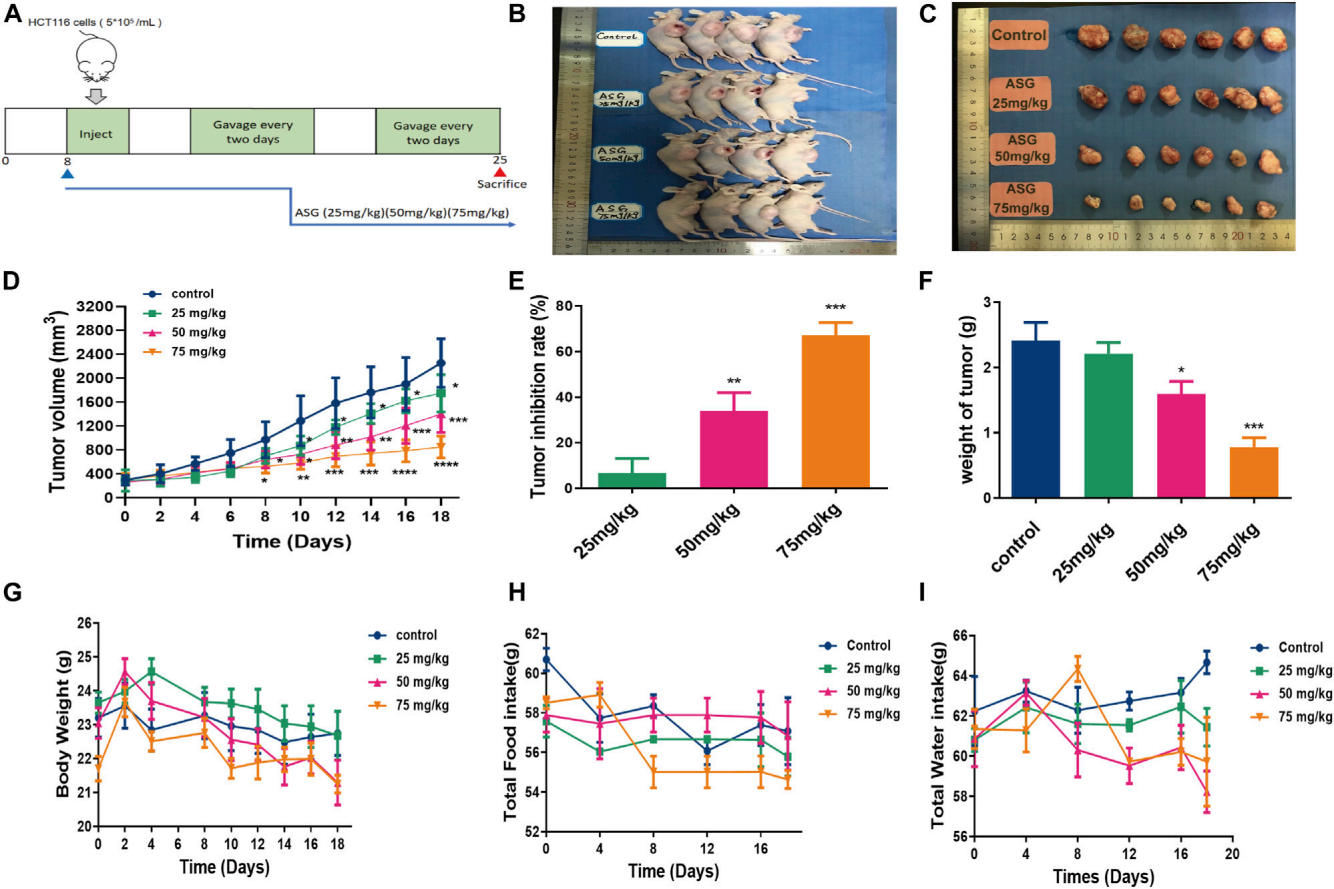
Astragalin decreased colon carcinogenesis in nude mouse models. **(A)** Diagram showing the experimental procedure for nude mouse models. **(B,C)** Astragalin decreased the size of HCT116 tumors. **(D)** Astragalin decreased the volume of HCT116 tumors. **(E)** Astragalin increased the rate of tumor inhibition. **(F)** Astragalin decreased the weight of HCT116 tumors. **(G)** Body weight, measured every 2 days. **(H)** Food intake, measured every 4 days. **(I)** Water intake, measured every 4 days. Data are expressed as mean ± SEM from six independent experiments (*n* = 6). *p < 0.05, **p < 0.01, ***p < 0.001 vs. Control.

**FIGURE 7:**
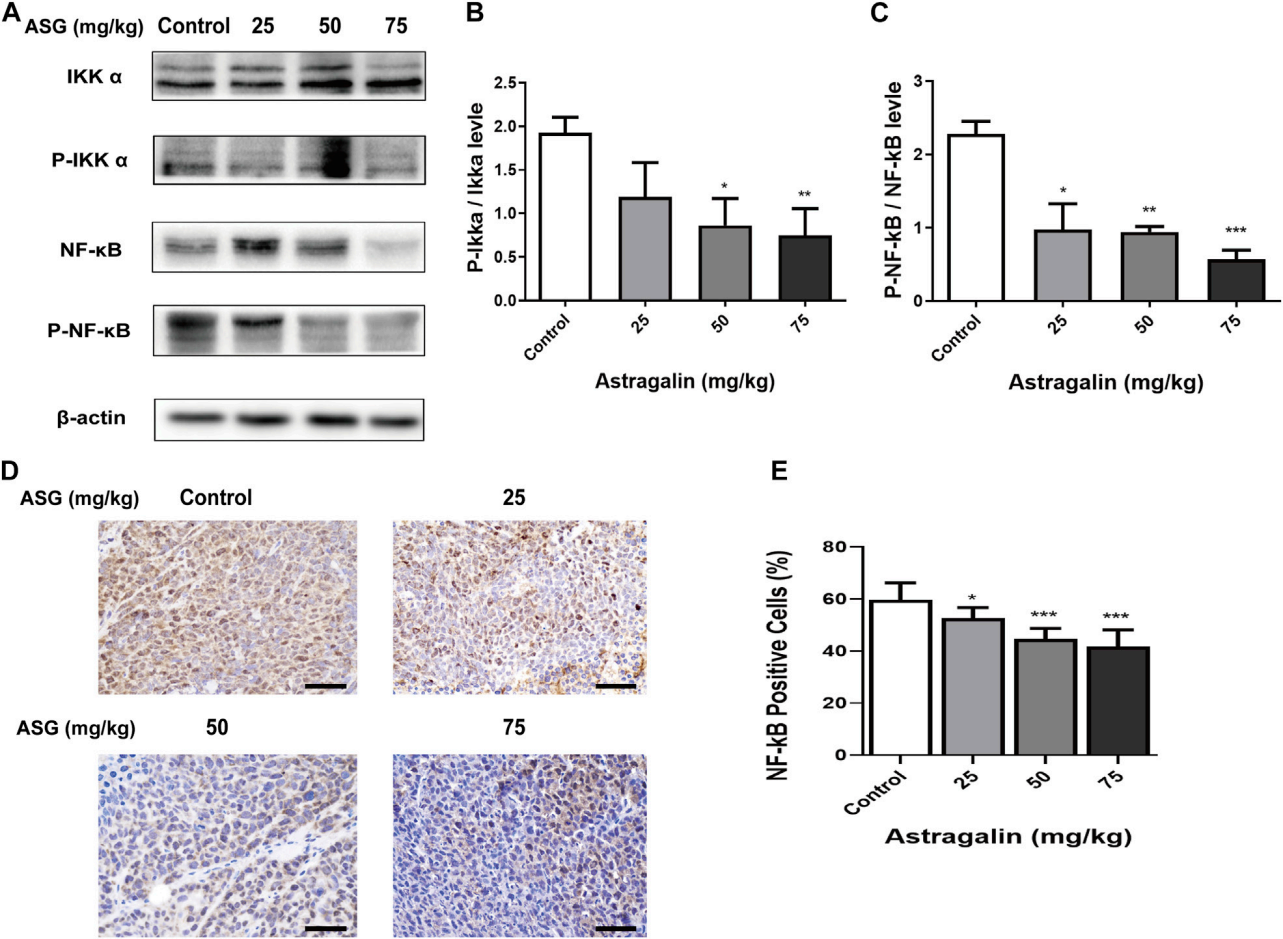
Astragalin regulated NF-κB signaling pathways in colon carcinogenesis nude mouse models. **(A–C)** Protein expression of Iκκα, p-Iκκα, NF-κB, and p-NFκB in colon tissues determined by western blotting. **(D,E)** NF-κB expression in colon tissues detected by immunohistochemistry. Data are expressed as mean ± SEM from three independent experiments (*n = 6*). *p < 0.05, **p < 0.01, ***p < 0.001 vs. Control.

In order to clarify the effects of astragalin on the development of colorectal tumors *in vivo*, we constructed a nude mouse model with subcutaneous xenotransplantation of human colon cancer. We measured tumor volumes, tumor weight and tumor formation rates of xenograft nude mice after treatment with astragalin, observed any changes in body weight and diet. The mice developed palpable tumors 14 days after subcutaneous injection of HCT116 cells. Mice were randomly divided into four groups and fed 0, 25, 50, or 75 mg/kg astragalin every 2 days by gavage. The weights and diet of the mice were recorded from the date of tumor calculation (Figure 6A). After sacrificing the mice, tumors were carefully removed; the tumor volumes of the astragalin groups were significantly smaller than those of the control group (Figures 6B–D). Compared with the control group, the tumor inhibition rates of the three treatment groups were 17.43%, 34.89% (*p* < 0.01), and 67.06% (*p* < 0.001) (Figure 6E). The mean tumor weights of the control group and the 25, 50, and 75 mg/kg treatments groups were 2.41 ± 0.11, 2.21 ± 0.13, 1.59 ± 0.15, and 0.77 ± 0.10 g, respectively (Figure 6F). At the end of the experiment, the average body weights of mice in the control group (without injection of HCT116 cells) and in the 25, 50, and 75 mg/kg treatment groups were 22.94 ± 0.12, 23.51 ± 0.15, 22.73 ± 0.21, and 22.16 ± 0.08 g, respectively. There was no significant change in diet (Figures 6G–I). We further studied the molecular mechanism by which astragalin inhibited tumor growth in the HCT116 xenografts by using western bolting and immunochemical staining to detect the expression of NF-κB protein in tumor tissues from mice. Compared with the control group, the expression of p-NFκB and p-iκκα in the astragalin groups was decreased, but there was no significant difference at 25 mg/kg (Figures 7A–C). Besides, immunochemical staining showed that the expression of NF-κB decreased with increasing drug dose (Figures 7D,E). These results indicate that astragalin might inhibit the development of colon cancer by inhibiting the expression of components of the NF-κB signaling pathway *in vivo*.

## Discussion

Flavonoids are secondary metabolites and bioactive compounds that exist widely in the plant kingdom (Rey et al., 2019). Astragalin is one of a variety of natural plant flavonoid substances found in *Moringa*, lotus leaf, tea, Chinese rose, and persimmon (Oldoni et al., 2019; Harikrishnan et al., 2020). Astragalin exhibits various pharmacological properties such as confer autophagy resistance and have anti-inflammatory, antioxidation, anti-apoptotic effects (Luo et al., 2010; Deepa et al., 2013; Li et al., 2013; Cho et al., 2015) and inflammation (Weiland et al., 2000; Kim B. W. et al., 2008), apoptosis, etc. For instance, astragalin could reduce the endoplasmic reticulum stress response and cell apoptosis, and relieve varicose veins caused by oxidative stress in rats to adjust abnormal sex hormones (Karna et al., 2019), and alleviate injury caused by UVB via the P38-MAPK pathway in an aging model (Li et al., 2018). Astragalin isolated from mulberry leaves by glycosides could reduce hemolysis of red blood cells induced by oxygen free radicals (Choi et al., 2013). As auxiliary components, astragalin and dihydromyricetin can enhance the protective effects of histidine, tryptophan, and ketoglutarate during cardiac arrest (Wang et al., 2018). However, the anti-tumor activity of ASG and their main mechanism have rarely been reported. In this study, we found that astragalin inhibited the growth and migration of human colon cancer HCT116 cells, suggest that it may be a promising anti-cancer for colon cancer. Therefore, the extraction of a high concentration of astragalin from natural plants as anti-cancer agent may become meaningful research in the future.

Astragalin exhibited low toxicity to normal cells. Astragalin inhibited the growth of hepatocellular carcinoma cells HepG2 cells, Hun-7 cells, H22 cells, but is less toxic to normal human untransformed hepatocytes cells HL-7702 cells (Li W. et al., 2017). Astragalin showed the anti-cancer effect on different kidney cancer cell lines Caki-1, A549, 786-O and 769-P by inducing cell apoptosis, but is less toxic to normal kidney cells hTRET (Zhu et al., 2019). In addition, astragalin isolated from the aerial part of *Delphinium Staphisagria* inhibited the proliferation of human leukemia cells HL-60 and HL-60/Bcl-x_L_, but didn’t impart any toxicity in normal human quiescent lymphocytes PBMC (Burmistrova et al., 2011). Furthermore, studies have shown that astragalin at a dose of 25–75 mg/kg had a significant inhibitory effect on LPS-induced inflammatory response (Soromou et al., 2012). Our previous research also found that astragalin at a dose of 50–100 mg/kg had a significant inhibitory on effect on DSS-induced ulcerative colitis by regulating gut microbiota and NF-κB pathway (Peng et al., 2020), and there was no apparent toxic effect on mice in this range of doses. In this study, we also found that astragalin had a significant inhibitory effect on different kinds of human colon cancer (HCT116, LoVo, SW620, SW480, Caco2), especially in HCT116 cells, and did not impart any toxicity toward normal human colon epithelial cell lines NCM460. Interesting, astragalin at the dose of 25–75 mg/kg had a significant inhibitory effect on the xenograft nude mouse tumor size and no significant effect on weight. The result suggests that astragalin may suppress the tumor growth of HCT116 cells, and exhibit no toxicity on the nude mouse.

Apoptosis is one form of programmed cell death. Apoptosis removes the defective or cancer cells and also prevents the development of drug resistance in cancer cells (Koff et al., 2015). Caspase 3 is the core of the protease activation cascade with an important role in the process of apoptosis (Weiland et al., 2000), activated caspase 9 then activate executioner caspase 3, caspase 6, caspase 7 and caspase 8 to perpetrate cell death at the minute (Parrish et al., 2013; Koff et al., 2015). The Bcl family proteins include anti-apoptotic Bcl-xl, Bcl-2, Bid, Bak, and Bax proteins that regulate apoptosis by effects on mitochondrial membrane permeability. Upregulation of the expression of pro-apoptotic proteins such as Bax and downregulation of anti-apoptotic proteins such as Bcl-2 can prevent apoptosis of cancer cells (George et al., 2009). Burmistrova et al. used astragalin to induce cell death in human leukemia cells by regulating the MAPK pathway, and observed significant changes in the activation of caspases and the Bax/Bcl-2 ratio (Burmistrova et al., 2011). This study found that astragalin inhibited the HCT116 cells proliferation in a dose-dependent manner by MTT assays and colony assays. Moreover, the cell cycle is an essential factor of cell growth; the cell cycle is regulated by complexes of cyclins and cyclin-dependent kinases during cell division (Lim and Kaldis, 2013), Cyclin D1 (Liu Y. et al., 2016), CDK1, CDK2, and CDK4 are often upregulated in tumor cells (Lee et al., 2011; Cho and Park, 2013). In the regulation of cell cycle phase, the nets work of cyclin-dependent kinases (CDKS) plays an important role. Cyclin E is a key regulator involved in the G1/S phase of the cell cycle (Xie et al., 2020). P21 and P27 were cyclin-dependent kinases inhibitors (CDKIS) that inhibited the activity of CDKS (Lee et al., 2011). P53 is an important anticancer protein and also repairs the cell genes. P53 could target p21 with subsequent cell cycle arrest and induced cell apoptosis (Xu et al., 2021). Therefore, in this study, we investigated the effect of astragalin on cell apoptosis and cell cycle on HCT116 cells. Western bolting showed that astragalin activated the expression of caspase 3, caspase 6, caspase 7, caspase 8, caspase 9, and P53, astragalin also significantly promoted Bax expression while suppressed Bcl-2 expression. Moreover, astragalin treatment decreased the expression of Cyclin D1, Cyclin E, and CDK2, CDK4, while enhanced the expression of P21 and P27. The cell growth was blocked in the G0/G1 phase, indicating that the anti-proliferation effect of astragalin on colon cancer cells may involve inhibition of apoptosis and cell cycle progression. Tumor metastasis and invasion are relatively complex and dynamic processes under the control of multiple factors.

Cell migration plays an important role in many physiological processes, such as tissue repair and tumor cell growth. In this study, astragalin significantly inhibited HCT116 cells migration by using the wound healing and transwell assays. MMPs have an important role in these processes, and are among the most critical regulatory molecules in cancer invasion and metastasis (Zucker and Vacirca, 2004; Choudhury et al., 2017). More than 20 members of the MMP family have been identified (Niknejad et al., 2012). Expression of MMP-2 and MMP-9 is highly negatively correlated with the occurrence and development of tumors (Song et al., 2016), and invasive osteosarcoma cell lines secrete large amounts of these proteins (Liu B. et al., 2016) also reported that MMP-2 and MMP-9 expression was significantly increased in tumor tissues of breast cancer patients. Our results are consistent with it, and ELISA assays showed that astragalin inhibited cancer cells’ migration by promoting the downregulation of MMP-2 and MMP-9 expression in colon cancer cells.

It has been increasingly recognized that the tumor microenvironment plays an important role in carcinogenesis. Inflammation often exists in the tumor microenvironment and is induced by inflammatory mediators such as TNF-α, IL-6, IL-1β (Silva et al., 2020). Many studies have shown that inflammatory cytokines are overexpressed during the progression of cancer. IL-6 powerfully stimulating the activation of the JAK/STAT3 pathway to promote tumor proliferation, motility, and invasion (Yoon et al., 2012). IL-1β induced significant upregulation of pro-inflammatory cytokines in astrocytic (Gajtko et al., 2020). TNF-α is a major factor involved in inflammation-associated cancer and plays an important role in the spread and invasion of tumor cells. Cancer cell activation also can be induced by numerous agents, including TNF-α (Zhang L. et al., 2020). This study evaluated the inhibitory effect of astragalin on TNF-α-induced in colon cancer HCT116 cells activated. The results showed that TNF-α could activate HCT116 cells in a short time, astragalin significantly inhibited the expression of TNF-α by using Elisa kit. These results indicated that astragalin inhibited HCT116 cells growth and migration through inhibition the production of inflammatory cytokines.

In recent years, the incidence of colon cancer in China has increased with improvements in living standards and diet changes (Pang et al., 2017). There are many types of colon cancer, and the most common of which is HCT116 (Huang et al., 2019). Prophase inflammation, which is commonly seen in malignant tumors, including breast and liver cancer, may induce cancer by causing abnormal activation of the NF-κB signaling pathway (Karin, 2006; Shen and Tergaonkar, 2009). NF-κB as a transcription factor is commonly upregulated in cancer cells and increases inflammatory cytokines (Liu et al., 2020). The NF-κB pathway exists widely as a transcription factor in the mammalian cell nucleus and is composed of five subunits (Hacker and Karin, 2006). NF-κB usually exists in the form of P65 and P50 dimers bound to inhibitory protein IκB in a non-functional state (Li et al., 2012). When cells are exposed to inflammatory stimuli, IκB is phosphorylated by the activated IκB kinase complex Iκκ and separates from NFκB, allowing NFκB to migrate into the nucleus and activate signaling pathways to promote the development and metastasis of cancer cells (Basseres and Baldwin, 2006). NF-κB activity largely controls the decision between proliferation and apoptosis with TNF-α treatment, suppressing the TNF-α-induce NF-κB pathway can potentiate TNF-α-induce apoptosis (Karin, 2006). Thus, to further clarify the mechanism of astragalin in HCT116 colon cancer cells, we examined the expression of NFκB cell signaling-related factors in astragalin-treated cells and nude mice *in vitro* and *in vivo*. In this study, we showed that astragalin concentration-dependently inhibited the expression of NF-κB phosphorylation and the subsequent downstream signaling mediators such as IκB, Iκκ, and NF-κB P65. Previous studies have demonstrated that astragalin could increase the sensitivity of lung cancer cells to apoptosis by regulating caspase-dependent and NF-κB signaling pathways, and reducing TNF-α- or LPS-induced inflammatory NF-κB pathways activation (Chen et al., 2017). Compare to this study, we did this work in combination with not only apoptosis but also with cycles. We also noted the effect of inflammatory cytokines on the tumor microenvironment. The result showed that astragalin also inhibited TNF-α induced NF-κB pathway activation. Furthermore, we inspected the NF-κB P65 translocation by using western bolting and immunofluorescent staining. The results showed that astragalin enhanced NF-κB P65 nuclear translocation and transcriptional activity in a time-dependent manner. These findings suggested that astragalin blockade of TNF-α/NF-κB signaling might be related to repressing migration and invasion in HCT116 cells.

Moreover, astragalin could significantly inhibit the formation and growth of xenograft tumors in nude mice in the present study. The results showed that treatment with astragalin significantly reduced the proliferation of the xenograft tumors, induced the apoptosis of tumor cells, and downregulated the NF-κB signaling pathway, consistent with our experiments *in vitro*. Furthermore, according to the studies of Li et al., who inhibited the growth of liver cancer cells using astragalin (Li W. et al., 2017) and Jia et al., who inhibited collagen-induced arthritis in mice using astragalin (Jia et al., 2019), the dose of astragalin used here was in the safe range. Whether the blocked TNF-α/NF-κB signaling and the intermolecular binding of astragalin to HCT116 cells was due to the feedback regulatory mechanisms needs to be verified in further study.

In conclusion, we found for the first time that astragalin treatment caused growth stagnation of intestinal cancer cell lines HCT116, whereas it had no toxic effect on normal colonic epithelial NCM460 cells. It was showed that astragalin had inhibited the proliferation and migration of HCT116 cells. These effects might be associated with the inhibition of apoptosis, cell cycle, and MMPs system. Molecular mechanism showed that the NF-κB signaling pathways were disrupted by astragalin in a dose-dependent manner *in vitro*, astragalin also inhibited the NF-κB P65 protein nuclear translocation stimulated with TNF-α. Furthermore, *in vivo* experiments showed that gavage with astragalin could reduce the proliferation of xenograft HCT116 tumors transplanted into nude mice, and decreased HCT116 tumor growth *in vivo* was related to upregulation of apoptotic cells in tumor tissues and inhibition of the NFκB signaling pathway. Finally, it is strongly suggested that astragalin has significant anti-inflammatory and anti-cancer effects, and may represent a new cancer treatment strategy. It is of important significance for astragalin applications as a new functional food ingredient and adjuvant in the clinical treatment of colon cancer. However, further *in vivo* pharmacological and clinical studies are needed to provide more reliable data to further clarify the mechanism by which astragalin exerts its effects on colon cancer cells.

## Data Availability

All datasets generated for this study are included in the article/Supplementary Material.
